# Does health-related poverty publication landscape reflect global needs in the light of the current poverty rebound?

**DOI:** 10.1186/s12992-022-00828-2

**Published:** 2022-03-21

**Authors:** Doris Klingelhöfer, Markus Braun, Dörthe Brüggmann, David A. Groneberg

**Affiliations:** Institute of Occupational, Social and Environmental Medicine, Goethe University, Theodor-Stern-Kai 7, 60590 Frankfurt, Germany

**Keywords:** Impoverishment, Inequality, Malnutrition, Healthcare, Famines, Research patterns

## Abstract

**Background:**

After nearly a quarter-century of declining poverty, the numbers are rising again significantly. This is due not only to effects of climate change but also to the COVID-19 pandemics and armed conflict. Combined with the enormous health impacts, that will cause misery and health care costs worldwide. Therefore, this study provides background information on the global research landscape on poverty and health to help researchers, stakeholders, and policymakers determine the best way to address this threat.

**Results:**

The USA is the key player, dealing mainly with domestic issues. European countries are also involved but tend to be more internationally oriented. Developing countries are underrepresented, with Nigeria standing out. A positive correlation was found between publication numbers and economic strength, while the relationship between article numbers and multidimensional poverty was negatively correlated.

**Conclusions:**

These findings highlight the need for advanced networking and the benefits of cross-disciplinary research to mitigate the coming impacts.

## Background

After more than 20 years of decline, poverty is currently on the rise again globally. Due to the increasingly threatening effects of climate change, 132 million people are expected to be pushed into poverty by 2030 as they live in regions at high risk of flooding [1].

Together with the 2019 pandemic of coronavirus disease (COVID-19), social and private costs are rising significantly while living standards are falling, plunging hundreds of millions of people back into poverty, not only in economically weak countries. There is evidence that inequality is also increasing in many parts of the world. The already poor and vulnerable people are the most affected by pandemic-related deprivation worldwide. COVID-19 has placed about further 120 million people in poverty, and that number is expected to rise to about 150 million by the end of 2021 [1–3].

In addition, the global impact of armed conflict is contributing to the rise in poverty, such as the conflicts in the Republic of Syria, which have caused extreme poverty to double in 3 years [1].

All of these consequences will have extreme impacts on the health, life expectancy, and rising mortality of millions of people. In addition to the coming humanitarian disaster situation, the associated costs will place an immense burden on healthcare systems around the world.

But what does poverty actually mean and how is it defined? In 2013, the World Bank defined extreme poverty as living below $1.90 per day, which primarily affects young people who work in agriculture and have little or no education. The “new poor,” as the World Bank puts it, will live more in cities, work less in agriculture, and instead work more in sectors affected by lockdowns or restrictions [3].

Other definitions of poverty relied on the inclusion of multiple indicators. Three key indicators of poverty can be identified: Health, education, and standard of living [4]. When these key dimensions are combined and aggregated, a more comprehensive and meaningful picture of poverty is obtained. The Multidimensional Poverty Index (MPI) announced annually by the Oxford Poverty and Human Development Initiative (OPHI) and the Human Development Index (HDI) of the United Nations are oriented accordingly [5].

The health dimension is based on life expectancy at birth. It includes the indicators nutrition and infant mortality. Caused by poor living conditions, such as insufficient access to healthy food, drinking water or medical care, poverty-related diseases threaten the health and life expectancy of millions of people. Especially children are suffering from the spread of infections or diarrhea as well as neglected tropical diseases that can be controlled in industrial countries [6]. Research and development (R&D) spending on poverty-related childhood diseases is judged to be far too low because of the perceived lower profitability [7]. When considering these aspects, one commonly thinks of the burdens on countries with low or medium economic power. That is why the multidimensional indices are developed specifically for developing countries. In reality, however, many people live in poverty even in rich nations [8].

Major efforts from a variety of disciplines are needed to address these issues. Scientists must be able to assess and evaluate the regional situation by creating appropriate simulation models that identify the most affected nations. In doing so, many and very different influences have to be taken into account. The expected increase in poverty-related diseases requires the readiness and commitment of all stakeholders, while current COVID-19 responses tend to neglect poor and focus on privileged [9].

Much work has already been done, but changing circumstances require rapid adaptation to the coming threats. The already elusive target of a global poverty rate of 3% by 2030 will be almost impossible to achieve without adequate attention. A large deficit in expenditures for R&D has been proven for poverty-related diseases with significant disparities between the individual poverty-related diseases [10]. The synergy effect of broad international networking seems to be important for this. That has already been demonstrated at the impact of research on poverty-related diseases as international European-Sub-Saharan-African collaborations. However, intra-Sub-Saharan African collaborations showed significantly less impact [11].

The publication figures per country and the related citation figures show the research effort with which a scientific question is dealt with. Regional conditions, interests and requirements form the focus of research planning and funding. Therefore, this study aims to provide an analysis of the global publication landscape on health-related poverty (P&H) to assess necessities for research under current circumstances and identify imperatives for future generations. The objective of mapping publication patterns by combining bibliometric characteristics and their relationship to geographic poverty burden will help research decision makers and stakeholders assess how global research is distributed according to regional needs and demands. In this way, multidisciplinary strategies can be developed to counter the effects of poverty-related health harms.

## Results

A total of 5527 publications (n) could be found by applying the search terms. Of these, most (56.32%) were original articles. Editorial material and meeting abstracts were published in about 13% of all publication types. Book reviews or chapters, letters, proceedings papers, reviews, and news items were published in only single digit numbers. Other document types were only sporadically present.

### Chronological analyses

The first article on P&H listed in the WoS was published in 1901. After the early years of P&H research, there was an annual publication output of less than ten articles until 1965. It was not until the mid-1960s that the number increased to double digits but then with a steady slope and a first peak in 1970 with *n* = 42 articles. In 2000, the number of P&H-related articles rose to a three-digit number for the first time. A first major peak in article counts was reached in 2007 (*n* = 289). This was followed by a lower performance period that ended in 2011 and led to another apparent peak in 2016 (*n* = 301). The year with the most articles published to date was 2020 with *n* = 311. In general, the development of citations received per year increased similarly to the number of articles published, with the exception of the years after 2010, when a clearly visible decrease could be observed. In terms of citations, there are some clear peak years. The years 1970 (c = 381), 1991 (c = 1409), 1997 (c = 3092), 2003 (c = 4331), and 2007, as the year with the most citations up to the time of the evaluation, (c = 4829) are striking. After that, the years 2010 (c = 4575 and 2014 (c = 3052) show peaks, but at lower levels. The sharp decline in citation numbers in recent years is due to the cited-half-life of citation numbers, which shows that publications need a certain amount of time to reach maximum citation numbers. Thus, this is a methodological phenomenon that is not due to declining interest in P&H research.

**Fig. 1:**
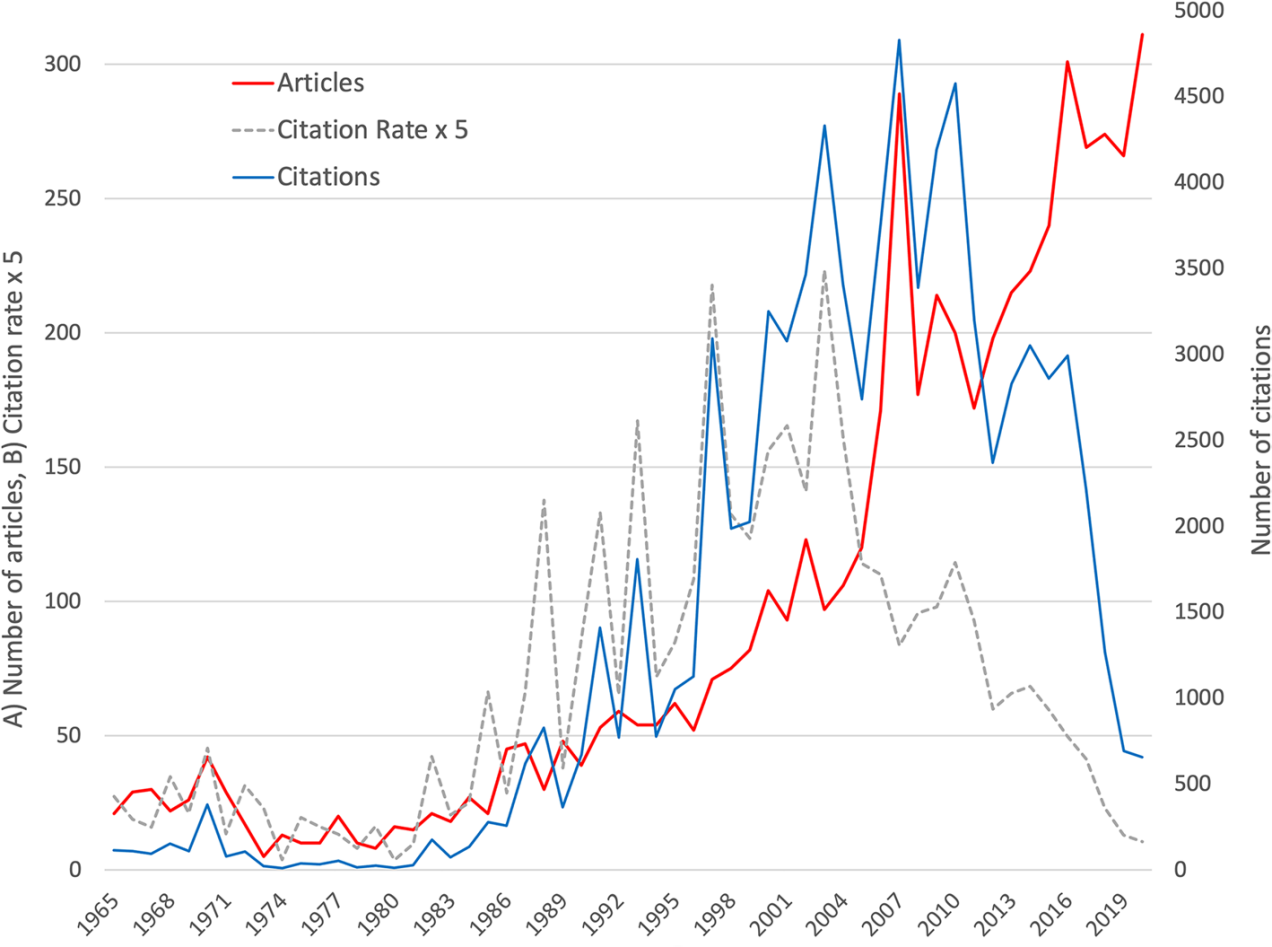
Development of the number of articles, the number of citations, and the citation rate (display: × 5) from 1965 to 2020

The years with the highest citation rates were 1997 (cr = 43.55) and 2003 (cr = 44.65), years with citation peaks but no exceptionally high publication output (Fig. 1).

The most frequently cited articles are listed in Table 1.

**Table 1:** Ten most-cited publications

Authors	Country	Year	c	Title	Source
Drewnowski, A. and Specter, S.E.	USA	2004	1440	Poverty and obesity: the role of energy density and energy costs	The American Journal of Clinical Nutrition
BrooksGunn, J. and Duncan, G.J.	USA	1997	1365	The effects of poverty on children	The Future of Children
Bangsberg, D.R. et al.	USA	2000	775	Adherence to protease inhibitors, HIV-1 viral load, and development of drug resistance in an indigent population	AIDS
Costello, E.J. et al.	USA	2003	631	Relationships between poverty and psychopathology - A natural experiment	JAMA
Diaz, R.M.	USA	2001	611	The impact of homophobia, poverty, and racism on the mental health of gay and bisexual Latino men: Findings from 3 US cities	American Journal of Public Health
Zenk, S.N. et al.	USA	2005	576	Neighborhood racial composition, neighborhood poverty, and the spatial accessibility of supermarkets in metropolitan Detroit	American Journal of Public Health
Patel, V. and Kleinman, A.	UK, USA	2003	534	Poverty and common mental disorders in developing countries	Bulletin of the WHO
Garmezy, N.	USA	1991	520	Resiliency and vulnerability to adverse developmental outcomes associated with poverty	American Behavioral Scientist
Lund, C. et al.	South Africa, Canada, UK	2010	490	Poverty and common mental disorders in low- and middle-income countries: A systematic review	Social Science & Medicine
Haan, M., Kaplan, G. A, and Camacho, T.	USA	1987	490	Poverty and health – prospective evidence from the alameda county study	American Journal of Epidemiology
Garmezy, N.	USA	1993	399	Children in poverty – resilience despite risk	Psychiatry
Patel, V., Rodrigues, M., and DeSouza, N.	India, UK	2002	398	Gender, poverty, and postnatal depression: A study of mothers in Goa, India	American Journal of Psychiatry

### Research foci

**Fig. 2:**
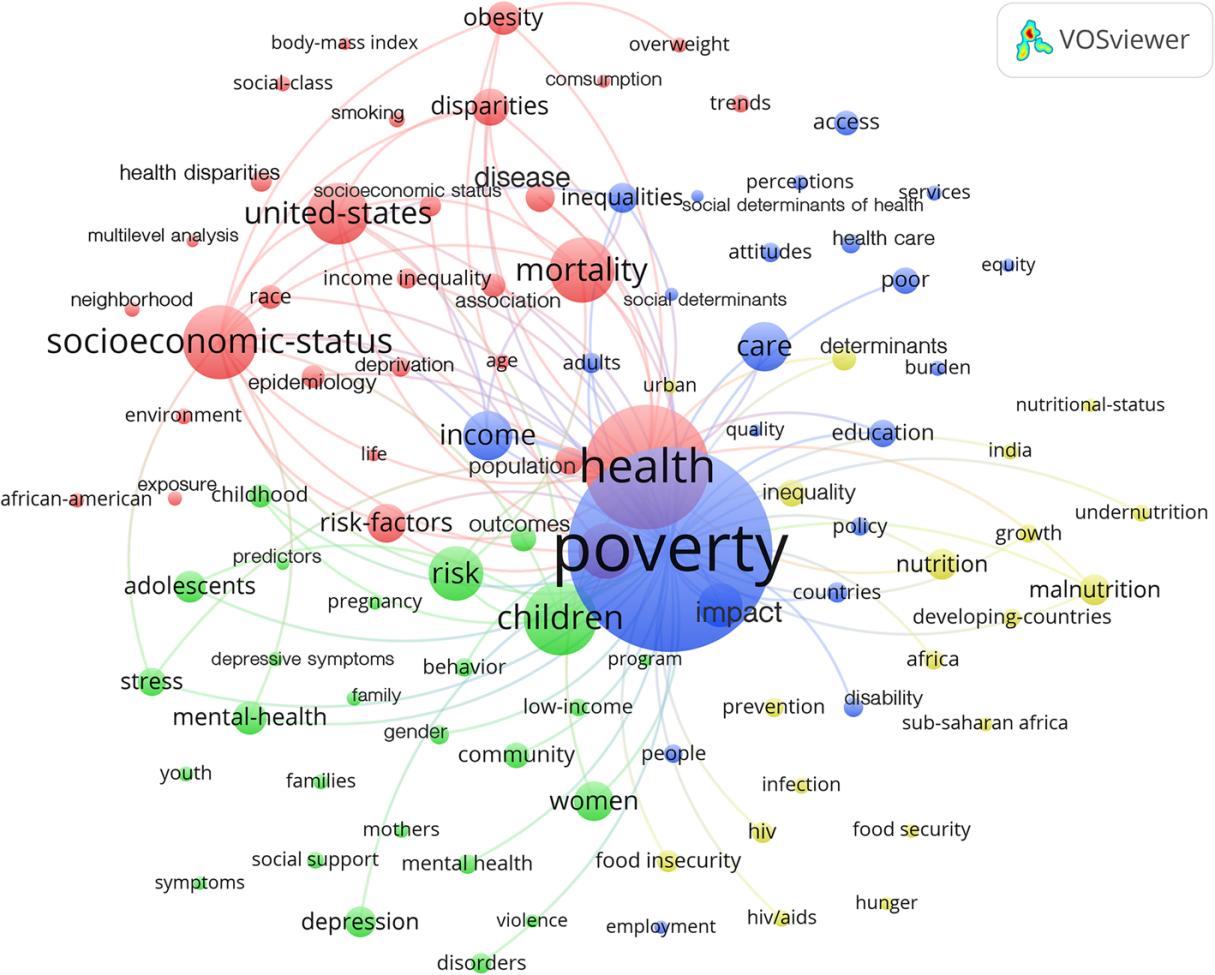
Cluster Analysis of the co-occurrence of keywords (threshold at least 35 occurrences), red cluster: Industrial countries (USA) and related burdens of poverty, green cluster: children and families and related burdens of poverty, blue: determinants and health care, yellow cluster: developing countries (Africa) and related burdens of poverty

The analysis of the thematical associations of the P&H research measured in the occurrence of keywords revealed four main clusters dealing with industrial and developing regions, children-related issues, and impact on income and health-care costs. Here, the USA as study area and the effects on children, adolescents, and families were quantitatively dominant (Fig. 2).

The sub-analysis of poverty-related diseases showed that AIDS was the most considered topic, to which *n* = 269 publications could be assigned. Tuberculosis was addressed in *n* = 99 publications and malaria in *n* = 59. Reference to children was evident in *n* = 1573 publications. Regionally, the USA was the most frequently mentioned study area with *n* = 532 publications. African countries were addressed in *n* = 417 publications.

### Geographic analyses

Out of all items, *n* = 4610 publication (83.40%) could be attributed to a country of origin by reading the collected metadata and thus included in the geographic analyses.

**Fig. 3:**
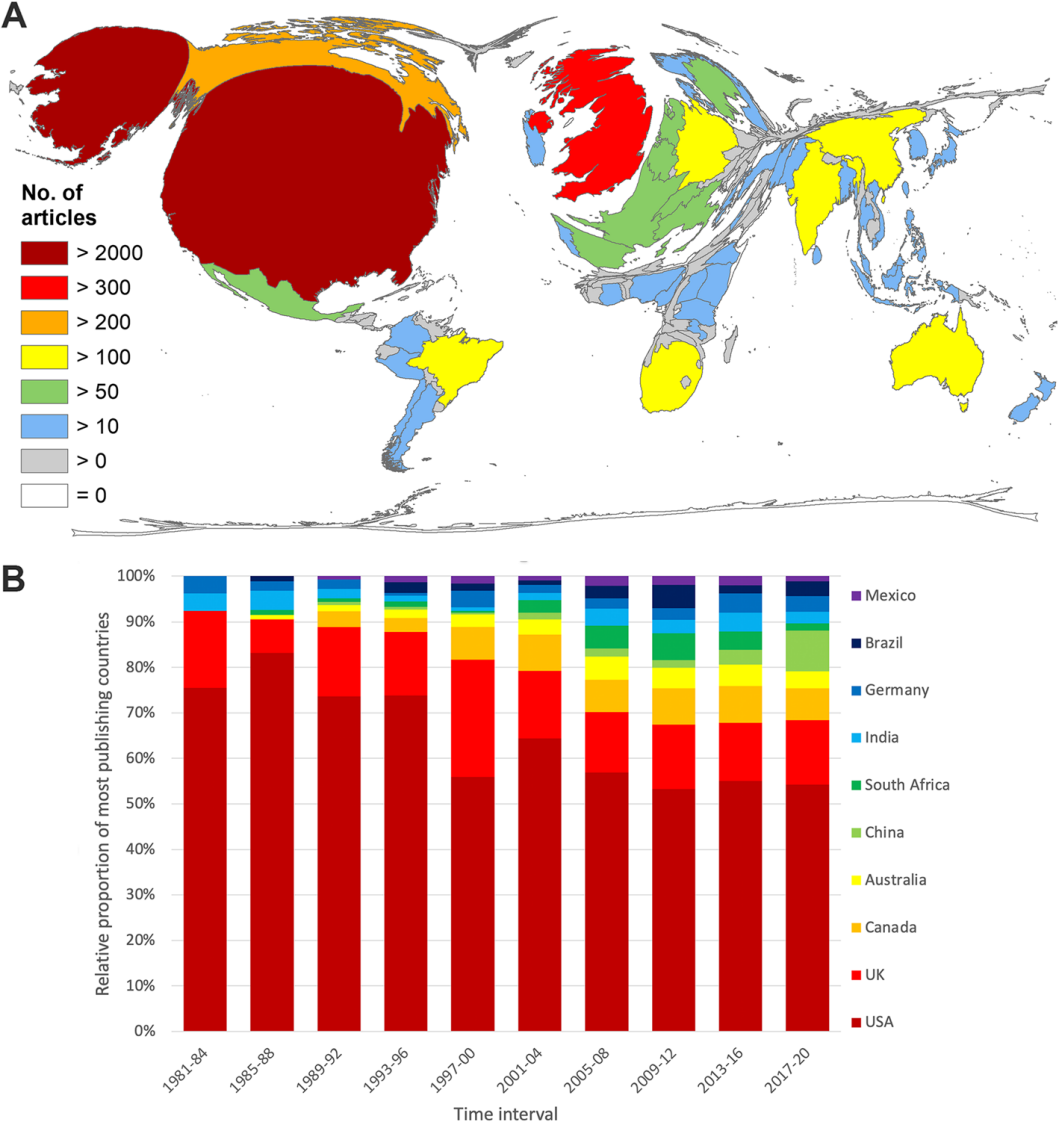
Geographical distribution of publication output on Poverty and Health (P&H). **A** Number of articles per country. **B** Development of the relative proportion of the most publishing countries on the publication output on R&H in 4-year intervals from 1981 to 2020

The country with the most publications on P&H was the USA with *n* = 2512 articles. The UK (*n* = 611) followed in second place with only about a quarter of the US-American articles. The next three ranks were occupied by Canada (*n* = 295), Australia (*n* = 164), and China (*n* = 155) as the first non-high-income-country in the evaluation. South-Africa, also an upper-middle-income country, followed in next place (*n* = 136). Brazil and Mexico, both emerging countries, ranked 9th and 10th (Fig. 3A). The African countries Nigeria and Kenya, as the most publishing lower-middle-income countries, ranked 19th and 21st, followed by the Asian lower-middle-economy Bangladesh in 22nd place.

**Fig. 4:**
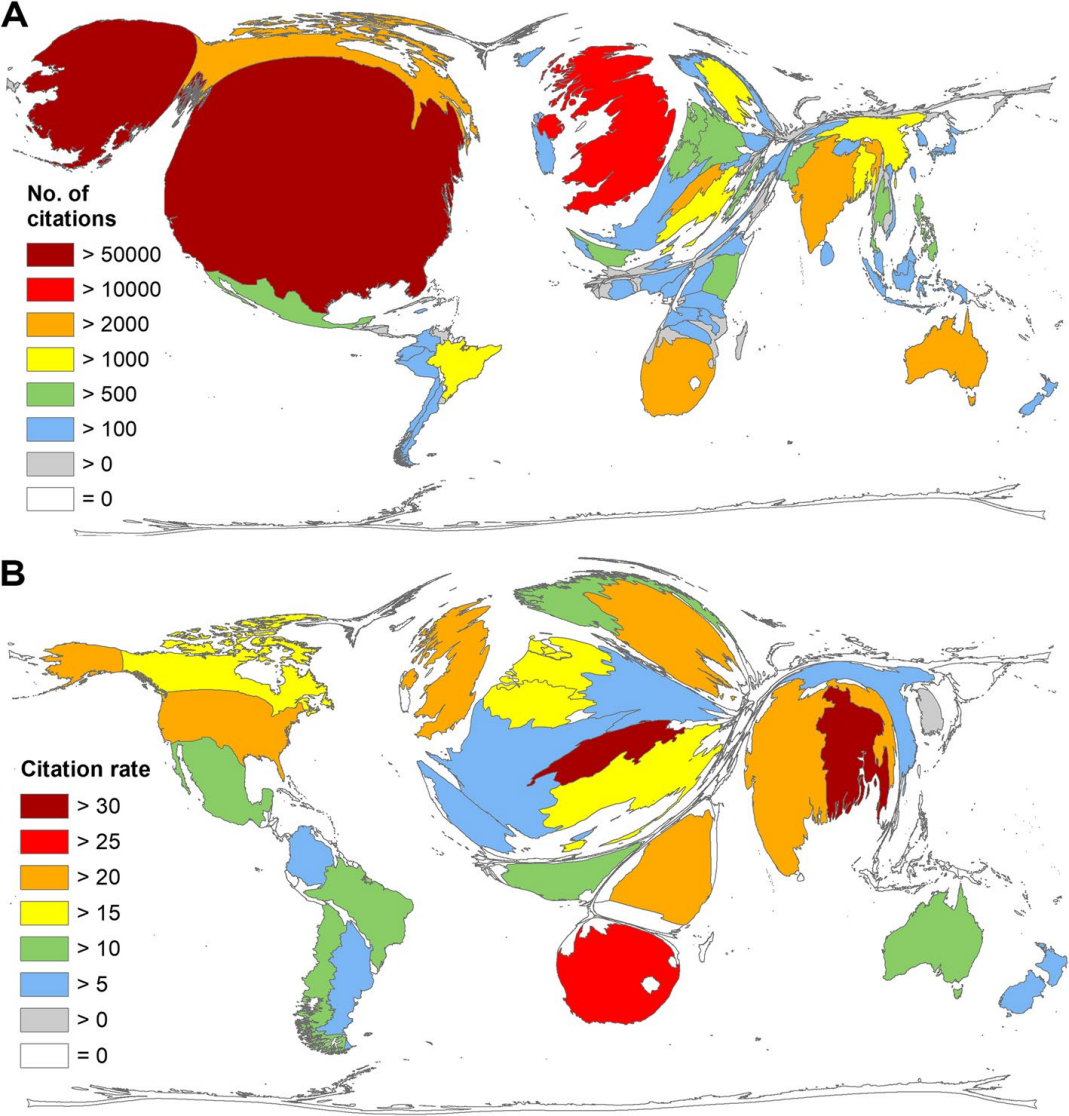
Citation parameters. **A** Number of citations per country. **B** Citation rate per country (threshold > 30 articles)

Analysis of the citations received at countries’ level revealed a similar picture of US-American dominance (c = 53,786), followed by the UK (c = 13,912). In contrast to the publication numbers, South Africa followed in 4th place (c = 3476) behind Canada (c = 4869). India reached 5th place (c = 2749). Switzerland, with only *n* = 62 articles on P&H, ranked 7th with its citation numbers (c = 2031) (Fig. 4A).

Switzerland’s performance on P&H was underlined by the analysis of citation rates (cr), which included 26 countries (analysis threshold = at least 30 articles on P&H per country). Here, it achieved a citation rate of cr = 32.76, ranking first. Bangladesh was also outstanding in terms of citation rate and achieving second place with cr = 31.68, followed by South Africa (cr = 25.56), the UK (cr = 22.77), and Kenya (cr = 22.48) (Fig. 4B). WHO was the most published institution based in Switzerland. Thus, the most cited study that was carried out in Asian countries, which also includes the co-authorship of Bangladesh, notes that out-of-the-pocket payments can exacerbate poverty [12]. The second most cited article is a systematic review that shows no conclusive effect of mental health interventions. It was first written by South Africa’s University of Cape Town in collaboration with WHO and others [13].

### International networking

**Fig. 5:**
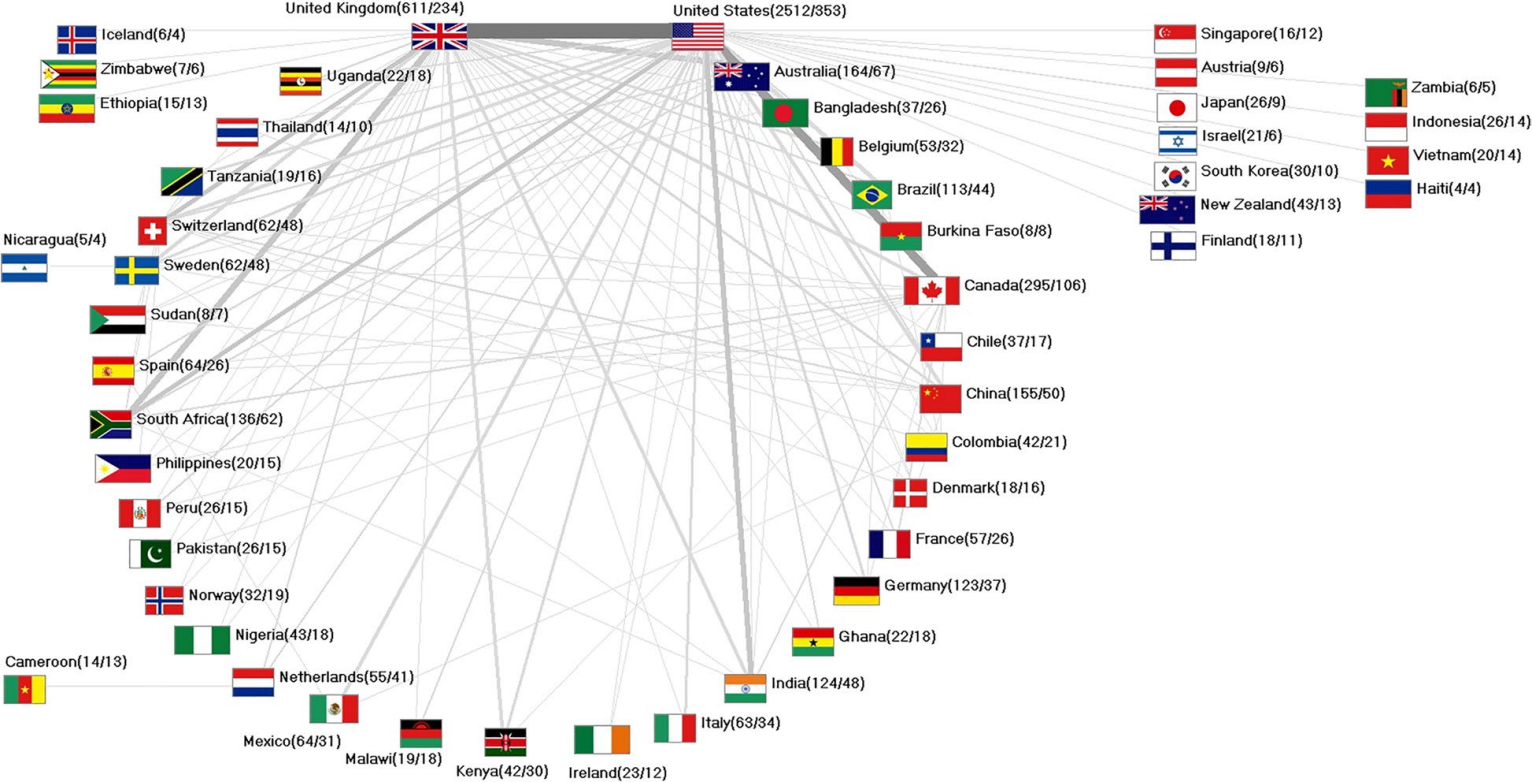
International network (display threshold at least 4 collaboration articles), numbers in brackets (number of publications / number of collaboration articles)

It was possible to identified *n* = 751 collaboration articles (16.29% of the geographically assignable articles). Of these, *n* = 546 articles were binational work, *n* = 126 articles were trinational, and the remainder were multinational collaborations. The most collaborations occurred between the USA and the UK (*n* = 78), followed by the USA and Canada (*n* = 41), the UK and Australia, tied with the UK and South Africa, as the first collaboration with a non-native English country (both *n* = 31). The developing country involved in the most collaborations was Kenya with *n* = 19 collaborations with the UK and *n* = 14 collaborations with the USA. Tanzania followed with *n* = 12 collaborations with the USA and Bangladesh with *n* = 11 US-American collaborations. Other low- or lower-middle economies integrated into the international network were Sudan, Nigeria, Ghana, Burkina Faso, Uganda and Malawi. On the bilateral side, Zimbabwe and Ethiopia (UK-collaborations), Zambia, Indonesia, Haiti (US-American collaborations), Nicaragua (Swedish collaborations), and Cameroon (Dutch collaborations) collaborated (Fig. 5).

At the national level, US-American institutions (mostly universities) were widely networked. At the international level, the University of London (UK) and the University of Cape Town (South Africa) reached *n* = 10 collaboration articles. These most frequently collaborating institutions also the ones that published the most.

### Socio-economic patterns

The GDP in 1000 bn US-Dollar and the total population of the publishing countries were related to the numbers of articles to provide deeper insight into national research efforts on P&H. Table 2 ranks the countries according to the two indices in order of the GDP-related parameter.

**Table 2:** Socio-economic ranking of the countries with at least 30 articles on P&H, *n* number of articles, *GDP* gross domestic product in purchase power parity (PPP) in current international $, *R*_*GDP*_ number of articles / GDP in 1000 bn PPP, *R*_*POP*_ number of articles / population in mill [14]. *HI* high-income country, *UMI* upper-middle-income country, *LMI* lower-middle-income country [15], ranked by R_GDP_

Country	*n*	GDP in 1000 bn PPP (current international $)	Population (mill.)	R_**GDP**_	Rank GDP	R_**POP**_	Rank POP
New Zealand	43	0.22	4.78	198.97	HI 1	8.99	HI 2
UK	611	3.26	67.53	187.68	HI 2	9.05	HI 1
South Africa	136	0.76	58.56	178.66	UMI 1	2.32	UMI 1
Kenya	42	0.24	52.57	177.16	LMI 1	0.80	LMI 1
Canada	295	1.93	37.41	152.86	HI 3	7.89	HI 3
Australia	164	1.35	25.20	121.26	HI 4	6.51	HI 6
USA	2512	21.37	328.24	117.52	HI 5	7.65	HI 4
Sweden	62	0.57	10.04	108.00	HI 6	6.18	HI 7
Switzerland	62	0.61	8.59	101.85	HI 7	7.22	HI 5
Norway	32	0.36	5.38	89.53	HI 8	5.95	HI 8
Belgium	53	0.63	11.54	84.61	HI 9	4.59	HI 9
Chile	37	0.48	18.95	77.61	HI 10	1.95	HI 11
Colombia	42	0.79	50.34	53.33	UMI 2	0.83	UMI 2
Netherlands	55	1.03	17.10	53.16	HI 11	3.22	HI 10
Bangladesh	37	0.81	163.05	45.84	LMI 2	0.23	LMI 2
Nigeria	43	1.07	200.96	40.01	LMI 3	0.21	LMI 3
Brazil	113	3.22	211.05	35.09	UMI 3	0.54	UMI 4
Argentina	35	1.03	44.78	33.94	UMI 4	0.78	UMI 3
Spain	64	1.99	46.74	32.20	HI 12	1.37	HI 13
Germany	123	4.66	83.52	26.40	HI 13	1.47	HI 12

New Zealand ranked first, followed by the UK, South Africa, Kenya, and Canada. The order changed in terms of populations: UK, New Zealand, Canada, USA and Switzerland. The correlations between the number of publications and the GDP in 1000 bn US-Dollars is highly significant (Pearson *p* < 0.0001). The correlation between the number of publications and the populations was also significant (Pearson *p* = 0.001).

### National poverty burden

The inclusion of poverty-related indicators also changes the ranking of countries. The multiplication of the GHI as a factor with the publication numbers (GHI(n)), shows India at the top with GHI(n) = 3856.4, followed by the USA (GHI(n) = 2512), which corresponds to the absolute publication number (all high-income countries were assigned GHI = 1 to include them in the analysis). On rank 3, South Africa was ranked (GHI(n) = 1972), followed by Nigeria (GHI(n) = 1337.3), and China (GHI(n) = 1178) (Table 3).

**Table 3:** Ranking of the 20 countries with the highest GH-normalized publication numbers, *GHI* Global Hunger Index, [16] *n* number of articles, high-income countries were assigned *GHI* 1

Country	Articles	GHI	GHI (Articles)
India	124	31.1	3856.4
United States	2512	1	2512
South Africa	136	14.5	1972
Nigeria	43	31.1	1337.3
China	155	7.6	1178
Kenya	42	23.2	974.4
Bangladesh	37	26.1	965.7
Brazil	113	8.5	960.5
Pakistan	26	32.6	847.6
Uganda	22	31.2	686.4
United Kingdom	611	1	611
Indonesia	26	21.9	569.4
Tanzania	19	29.5	560.5
Malawi	19	26.5	503.5
Ethiopia	15	29.1	436.5
Mexico	64	6.5	416
Philippines	20	20.2	404
Ghana	22	15.2	334.4
Colombia	42	7.7	323.4
Vietnam	20	16	320

**Fig. 6:**
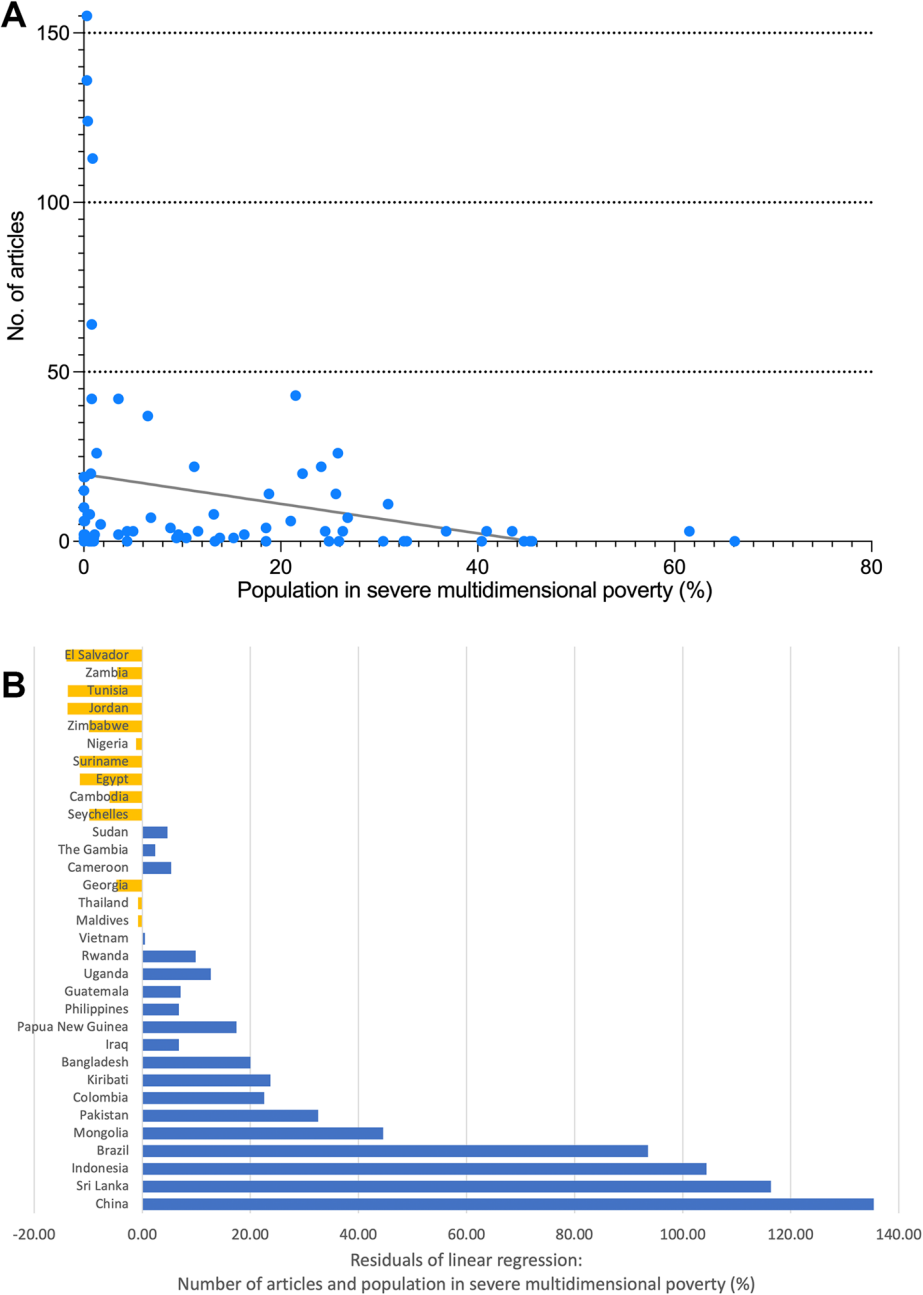
Association between number of articles and population living in multidimensional poverty (Multidimensional Poverty Index) [4] of developing countries. **A** Linear regression. **B** Residuals of 20 most publishing developing countries. Yellow: negative residuals, blue: positive residual

Meaningful correlation analyses were conducted between the number of articles of developing countries and the population living in multidimensional poverty according to the MPI [4]. A significant negative correlation (Pearson *p* = 0.054) was found. Figure 6A shows the linear regression of the two variables. The analysis of the residuals of the 20 most publishing developing countries listed China, Sri Lanka, Brazil, and Mongolia as countries with the highest deviation from the regression line in favor of the number of articles. While El Salvador, Tunisia and Jordan showed the highest deviation in the negative directions, albeit to a lesser extent. (Fig. 6B).

### Funding

A total of 2048 articles (37.05%) received 3472 grants. There were a variety of funders, ranging from governments, universities, hospitals, independent research institutions, non-profit organizations and foundations to private companies and banks. Also, international associations, societies and inter-governmental organizations participated in research funding on P&H (Table 4).

**Table 4:** Donors of P&H research

Donor	Grants
Governments	2379
Universities, Colleges	304
Foundations, Trusts	259
International Organizations, Programs	202
Companies	180
Associations, Societies	55
Hospitals, Health Centers	38
Independent Research Institutions	13
Banks	13
Non-profit Organizations, Charities	10
Non-Governmental Organizations	9
Churches, Religion-based	7
Journals, Databases	2
Museums	1

Governments of 51 countries (inclusive regional and local governments) granted funds for research on P&H (Table 5).

**Table 5:** Most funding governments, %*Grants* Grants/Articles × 100

Country	Articles	Grants	%Grants of articles
USA	2512	1665	66.28
Canada	295	154	52.20
China	155	137	88.39
UK	611	117	19.15
Australia	164	44	26.83
Sweden	62	28	45.16
South Africa	136	26	19.12
Brazil	113	25	22.12
Japan	26	17	65.38
Germany	123	16	13.01
Spain	64	12	18.75
New Zealand	43	9	20.93
Switzerland	62	9	14.52
Denmark	18	8	44.44
South Korea	30	8	26.67
India	124	7	5.65
Netherlands	55	6	10.91
Nigeria	43	6	13.95
Chile	37	5	13.51
Taiwan	13	5	38.46

A total of 2379 grants (g) were awarded by governments including international collaborations (g = 19). The USA was by far the largest funder of P&H research with 1665 grants (g). Of these, most grants (g = 1382) were awarded by the National Institute of Health (55.02%), with 1249 attributable to the respective institute. The National Institute of Child Health & Human Development (NICHD) funded g = 213 times, followed by the National Institute of Mental Health (NIMH) (g = 175) and the National Institute on Drug Abuse (NIDA).

The most funding foundations were The Wellcome Trust (UK), giving g = 88 grants, that were partly awarded together with programs from the European Union (EU), the Robert Wood Johnson Foundation (USA, g = 35), and the Melinda and Bill Gates Foundation (USA, g = 27).

## Discussion

The global landscape of P&H research, like many other research fields, is characterized by increasing publication numbers, disciplinarity, and international collaborations. The evaluation period of this analysis went back to 1900 as the earliest year indexed in WoS. Therefore, the first article included in the analysis database dates from 1901 [17], although the scientific interest in poverty research started already earlier. This article was published in the Journal of the American Medical Association (JAMA). Author N.S. Davis was a professor of medicine with a reputation as a “humanitarian reformer in medical and civic matters” who was an advocate for systematic aid to the poor and fought to establish a hospital for the sick poor. Even before this article on the causes of poverty and ways to avoid it, he was concerned with combating poverty, e.g., an 1870 publication in which he attributed relapsing fever “to the wants of poverty and destitution in crowded and badly ventilated, uncleanly habitations” [18]. He was the founder of the American Medical Association (AMA), which established the world’s first national code of medical ethics [19].

In the first decades of the twentieth century, the USA, in addition to some European efforts, devoted most of its attention to poverty research. The rise in engagement during this period was due in part to the rise of “neoliberalism,” which contrasted with the “laissez-faire” individualism of the industrial age in the United States and European political culture [20]. However, the number of publications remained in single digits until the mid-1960s. Liberalism, under the influence of the civil rights, welfare state, and women’s movements, led to controversial debates in the scholarly community during this period. That was when poverty became an analytical category divorced from racial inequality. Lyndon B. Johnson’s proclamation of the “War on Poverty” in 1964 certainly also contributed significantly to the increase in publication numbers during this period, with studies focusing mainly on the national level of the USA [20]. In the 1990s, the influence of NGOs was more noticeable with their efforts to alleviate poverty in the “Third World.” During this period, for example, the number of NGOs in the Philippines increased from a few hundred to more than 20,000, spurred by the government’s encouragement of funding for poverty research [21]. The annual World Bank Conference on Developing Countries in 1995 triggered a rethinking of the course of development and aid to the poor worldwide [22]. The formulated Millennium Development Goals (MDGs) additionally triggered a push for spending on poverty reduction in poor countries, thus also increasing publication numbers [23].

Nevertheless, research on poverty-related diseases has been and continues to be sparse compared to other topics. In particular, the number of studies from developing countries is small and published in journals with relatively low impact [23].

The early focus is on poverty in the USA and European countries conducting the research, which includes health issues related to the problems of the poor in affluent countries. In the USA, these were mainly problems of minorities. Family and child-related research, which grew over time, focused more and more on the psychological and mental effects. The most cited publications are mainly related to these topics, additionally highlighting the yearly peaks in the development of publications over time. 9 of the 10 most cited publications are from the United States. Only the Lund et al. (South Africa) article on mental disorders addresses the burden in developing countries [24]. Economic and epidemiological evidence from von Philipsborn et al. (2015) shows that poverty-related diseases are neglected in R&D spending. A list of 26 diseases was found to account for 13.8% of the global burden of disease but receives only 1.34% of R&D spending [10].

The USA provided the most funding for P&H research in this study, with the NIH being the major funder. Of the NIH institutes, the NICHD funds the most, which also shows the focus on child-related research. Therefore, it is not surprising that the USA ranks first in terms of publication output on P&H. In contrast to other scientific topics, where China in particular is approaching or even overtaking the USA [25–27], research on P&H has been clearly US-dominated to date. China has reached 5th place over time, although the gap with the US performance is still very large. A previous study of China’s research performance on global health found comparable results with China lagging behind. Global health is a is a relatively new focus there. For which there was not a single specialized institution a few years ago [28]. However, as the number of poor in China declined from 66.3% in 1990 to only 0.5% in 2016 (poverty headcount ratio at $1.90 a day [29]), the relative contribution to the linear regression line of articles and population in severe multidimensional poverty measured in the residuals indicated China as the country with the largest positive deviation. The overall results of this correlation analysis show the general negative relationship between countries’ publication output and the proportion of poor population, and thus the underrepresentation of developing countries in terms of absolute publication output. Regarding the associations between poverty parameters, only the relationship between the number of poor and the number of articles was significant (positively correlated) in terms of the absolute number of poor at the national poverty lines [29]. In contrast, the relationship between countries’ publication numbers and economic strength, as measured by GDP (in 1000 bn US-Dollars), is highly correlated. These results show how important economic strength is for global research performance. The indices applied to assess the performance of countries in P&H research must be seen against the background that key poverty indicators such as famine or hunger reflect only one side of the coin. Famines were caused in many cases by political oppression, corruption, or economic mismanagement, and this continues to account for most food insecurity to this day [16].

Countries with low economic power that could be highlighted by normalizing the GHI of the present study are Nigeria, Kenya, Bangladesh, Pakistan, and Uganda. An hot spot of impending poverty is Sub-Saharan Africa, where about one-third of citizens are expected to be at risk of poverty due to COVID-19 [1]. In the five countries of Nigeria, the Democratic Republic of Congo, Tanzania, Ethiopia, and Madagascar, nearly half of sub-Saharan Africa’s poor live [3]. Of these countries, Nigeria is the only one with a significant number of publications that puts it above the threshold used for the socioeconomic analyses in this study. Compared with all African countries, Nigeria was the most productive country after South Africa. The scientific output of these countries is also confirmed by another study looking at the publication performance of sub-Saharan countries in cardiovascular diseases [30]. This study also confirmed the relatively high percentage of domestic publications of the Nigerian studies, which was also noted in this evaluation. The analysis on funding for research also reflects the roles of South Africa and Nigeria in P&H research. South Africa awarded 26 grants and Nigeria 6 grants for P&H research. That makes them the only African countries with more than one financial contribution from governments. In Nigeria, research is mainly conducted by government research institutes and universities. Since Nigeria has the largest population in Africa and thus the largest number of students, the number of publications is also higher than in other West African countries [31]. A previous study analyzing publishing journals found large disparities between low- and high-income countries in the quality and quantity of research outputs in the biomedical and global health sciences because of scarce access to research and low government funding. That is also true for Nigeria [31].

An international network for P&H research has been established with the US and UK as the main partner countries, and unlike other research areas, it includes a relatively large number of developing countries, albeit at a comparatively lower level [32]. In comparison, the international collaborative landscape on diseases emerging in Africa, such as studies on Ebola [33] or Malaria [34] included a similar number of African countries, albeit in higher numbers. In the present study, however, the proportion of articles published in collaboration is significantly higher in developing countries than in higher-income countries, with the exception of Nigeria, which also has a ratio of about 40%, similar to the UK and other European countries. In contrast, the USA cooperated with other countries to a much lesser extent (14%). Compared to previous studies [32], however, the proportion of international collaborations in P&H research is lower in high-income countries. Previous studies also emphasize the benefits of working together in an inter- or multi-disciplinary and international manner as the only way forward for future poverty research [11, 35].

## Conclusions

This study focuses on global publication output on health-related poverty, incorporating advanced socioeconomic, poverty, and financial parameters. Under the pressure and threat of changing poverty figures worldwide, which are rising for the first time in nearly a quarter-century, scholars worldwide are challenged. Many nations have to be engaged and working in many different sectors to secure the lives and living standards of their population. Our findings highlight the lack of international collaborations and research approaches. In particular, research on poverty-related diseases is not sufficiently addressed. The research landscape is comparatively dominated by domestic studies. There is a big difference between developing countries and industrialized countries. Therefore, all industrialized countries, especially the USA as a major player, should further expand their scientific efforts to meet the needs of the new global situation. The most affected countries should be involved in collaborative networks to create a win-win situation, since poor countries find it difficult to fund adequate studies, while healthier economies benefit greatly from experience and regional expertise.

## Methods

### Data source and methodological platform

The Web of Science (WoS) online database was used as the data source. The core collection of this database ensures the inclusion of peer-reviewed scientific literature and the evaluation of citation-based parameters by the Science Citation Index Expanded (SCIE). WoS is one of the most recommended data sources for bibliometric analysis [36] and provides a broad selection of research papers published in internationally renowned journals. The database created thus forms a reliable and meaningful source for the evaluation of publications on the topic P&G and also enables the analysis of citation parameters. Citations are otherwise only available in Scopus and Google Scholar, and these sources are either time-limited or not rigorously peer-reviewed [37]. Therefore, WoS has proven to be the best choice for bibliometric studies.

WoS is the reference data source for all analyses carried out as part of the bibliometric platform “New quality and quantity indices in science” (NewQIS), the methodology of which also forms the basis of the present study [38, 39]. NewQIS combines the analysis of established bibliometric data, which characterize publication behavior according to temporal and geographical aspects, with topic-related values, which allow a more in-depth assessment of publication patterns. In addition, the NewQIS platform presents bibliometric findings for the first time using density equalizing map projections (DEMPs) based on an algorithm by Gastner and Newman [40] to create map cartograms for each analysis parameter. These cartograms were created with ArcGIS application. Cluster analyses of all keywords used were performed using the VOSviewer application [41] to show the occurrence and distribution of research foci.

### Search strategy

**Fig. 7:**
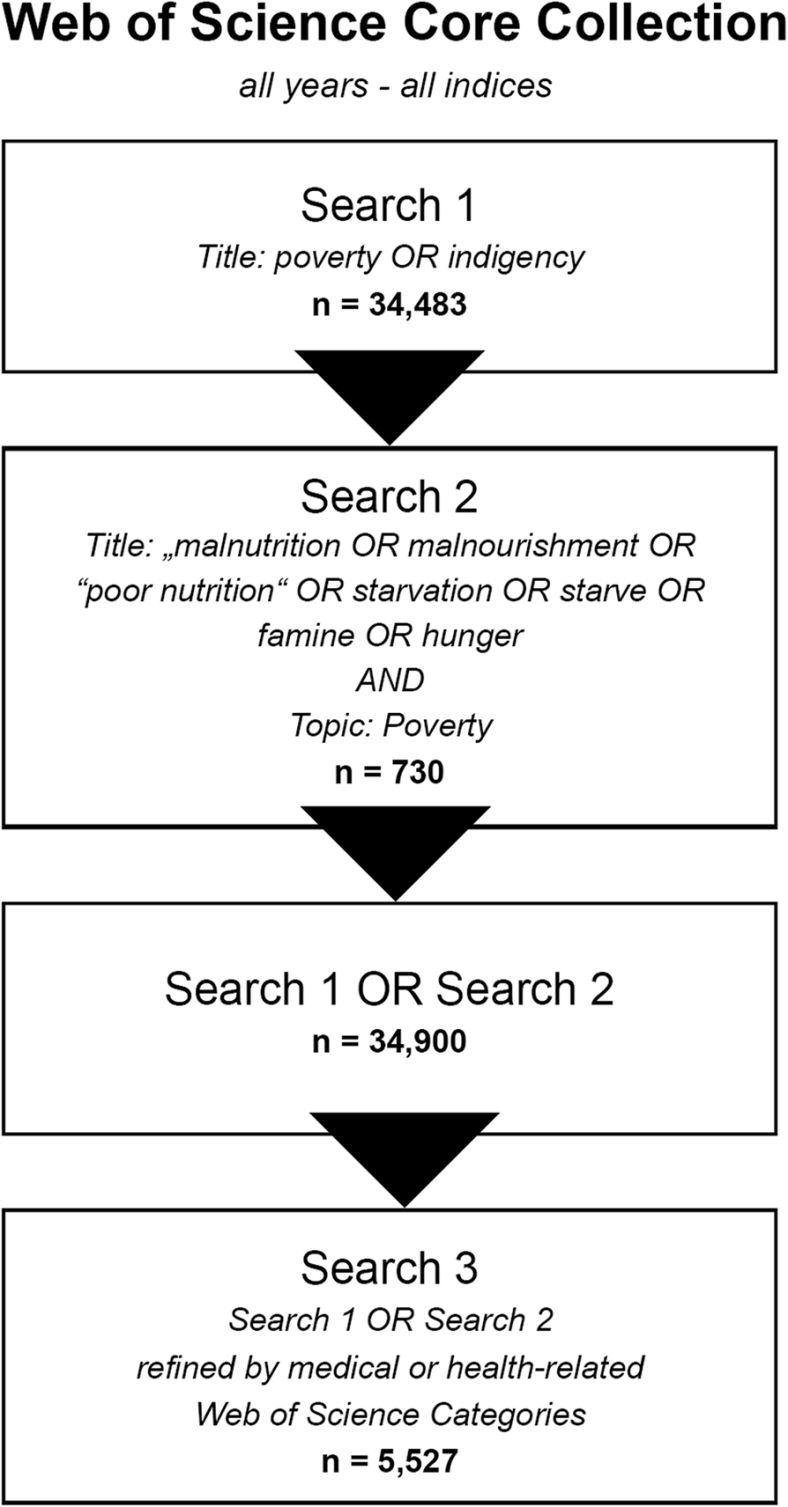
Search procedure

The WoS Core Collection search was performed on 05/20-21. The generation of a representative and valid database of metadata of publications in the field of P&H research was ensured by applying the following search term and strategy (Fig. 7):

1. Title search (“poverty” OR “indigency”)

OR

2) Title search (“malnutrition” OR “malnourishment” OR “poor nutrition” OR “starvation” OR “starve” OR “famine” OR “hunger”) AND Topic search (“poverty”). The second set of search terms had to be combined with the term “poverty” as they are not related to this topic on their own.
3) Subsequently, the entries retrieved were filtered by including only health-related WoS categories.

For the sub-analysis on poverty-related diseases, the search string was extended to include the three main diseases “malaria,” “HIV/AIDS,” and “tuberculosis,” as well as child-related topics. In addition, a regional sub-analysis was conducted that included both the USA and the publishing African countries.

The retrieved metadata were saved in an MS Access database and sorted according to the individual analysis parameters. Manual unification must be performed for non-standardized information on affiliations, authors, and subject areas.

### Evaluation parameters

This study is based on a variety of parameters that, when combined, provide a comprehensive and meaningful picture of the global P&H landscape. Established bibliometric parameters such as publication output, citation counts, citation rate, keywords, and socio-economic characteristics of the publishing countries are used to allow a more profound comparison.

Most publishing countries were sorted by their bibliometric data and evaluated according to the economy status of the World Bank’s lending category [15].

The socio-economic parameters used, total population (in mill. inhabitants) and gross domestic product (GDP in 1000 bn PPP current international $) are from the 2019 UIS Statistics as the most recent data set. Here, some country data also comes from 2018, as the 2019 data was not yet available [14]. Missing data, e.g., for Japan and the USA, were taken from the World Bank [42, 43].

Various values and indices were used to evaluate research effort by the need in relation to poverty levels. World Bank data were used to analyze the relationship between countries’ publication output and the proportion of people living in extreme poverty (poverty headcount ratio at $1.90 a day (2011 PPP) (% of population) [29]. For each country, the latest available data were used, with 2016 being the most recent year [44].

The Global Hunger Index (GHI) [16] combines the indicators: undernourishment, child wasting, child stunting and child mortality to estimate the burden of hunger worldwide. It is measured annually on regional and national levels. It ranges on a scale from 0 to 100 with 0 being the best index and 100 the worst [45]. Here, it was multiplied as a factor with the absolute publication numbers to obtain a hunger normalization for countries’ publication performance. The high-income countries that were not listed were given an index of 1 to include them in the evaluation.

One feature of the MPI [4] for developing countries, the percentage of the population living in severe multidimensional poverty was used for correlation analysis and linear regression with residuals.

For all ratio parameters, e.g., citation rate, a threshold of at least 30 articles on P&H per country was set to reduce bias from extremely low-publishing countries.

## Data Availability

The bibliometric data is owned by and has been obtained from the Web of Science database. Therefore, authors are not allowed to share the data publicly or privately. Any researcher with access to the Web of Science database can obtain the data using the methods described in the paper.

## Abbreviations

AMA: American Medical Association
DEMP: Density Equalizing Map Projection
EU: European Union
GHI: Global Hunger Index
GDP: Gross Domestic Product
HDI: Human Development Index
MDG: Millennium Development Goals
MPI: Multidimensional Poverty Index
NewQIS: New Quality and Quantity Indices in Science
NICHD: National Institute of Child Health & Human Development
NIH: National Institutes of Health
NIMH: National Institute of Mental Health
NIDA: National Institute on Drug Abuse
OPHI: Oxford Poverty and Human Development Initiative
P&H: Poverty and Health
PPP: Purchasing Power Parity
R&D: Research and Development
SCIE: Science Citation Index Expanded
WoS: Web of Science
